# Attention, cognitive control and motivation in ADHD: Linking event-related brain potentials and DNA methylation patterns in boys at early school age

**DOI:** 10.1038/s41598-017-03326-3

**Published:** 2017-06-19

**Authors:** Hartmut Heinrich, Juliane Grunitz, Valeska Stonawski, Stefan Frey, Simone Wahl, Björn Albrecht, Tamme W. Goecke, Matthias W. Beckmann, Johannes Kornhuber, Peter A. Fasching, Gunther H. Moll, Anna Eichler

**Affiliations:** 1 Dept. of Child & Adolescent Mental Health, University Hospital Erlangen, Friedrich-Alexander University Erlangen-Nuremberg, Erlangen, Germany; 2 kbo-Heckscher-Klinikum, München, Germany; 3 Research Unit of Molecular Epidemiology, Helmholtz Zentrum München, German Research Center for Environmental Health, Neuherberg, Germany; 4 Institute of Epidemiology II, Helmholtz Zentrum München, German Research Center for Environmental Health, Neuherberg, Germany; 5 German Center for Diabetes Research (DZD), Neuherberg, Germany; 6 Dept. of Child and Adolescent Psychiatry, University Medical Center Göttingen, Göttingen, Germany; 7 Dept. of Obstetrics and Gynecology, University Hospital Erlangen, Friedrich-Alexander University Erlangen-Nuremberg, Erlangen, Germany; 8 Dept. of Obstetrics and Gynecology, Medical Faculty, RWTH Aachen, Aachen, Germany; 9 Dept. of Psychiatry and Psychotherapy, University Hospital Erlangen, Friedrich-Alexander University Erlangen-Nuremberg, Erlangen, Germany

## Abstract

In order to better understand the underpinnings of attention-deficit/hyperactivity disorder (ADHD), we targeted the relationship of attentional, cognitive control and motivational processes with DNA methylation patterns of 60 candidate genes in boys at early school age. Participants (6 to 8 years; N = 82) were selected from a German longitudinal cohort (FRANCES). ADHD-related behaviour was assessed via maternal ratings. Performance and event-related potential measures (inter alia Cue-P3 and Nogo-P3), which were recorded in a motivational go/nogo task, indicated diminished attentional orienting, reduced inhibitory response control and a larger motivational effect on performance in ADHD already at this relatively young age. Methylation patterns were analysed in buccal cell DNA with the Illumina HumanMethylation 450K array. For CpG sites at genes of the dopaminergic (*COMT*, *ANKK1*) and the neurotrophic (*BDNF*, *NGFR*) system, associations with the Nogo-P3 as well as ADHD symptom severity were found suggesting that these systems are involved in response control deficits in ADHD. Methylation effects related to both functional aspects and ADHD behaviour were also observed for *DPP10* and *TPH2*. Epigenetic mechanisms may play a role in ADHD-associated deficits but findings need to be replicated in larger samples and are limited by the fact that only peripheral methylation could be considered.

## Introduction

Inattention, motor hyperactivity and impulsivity are the cardinal symptoms of attention-deficit/hyperactivity disorder (ADHD), one of the most prevalent child psychiatric disorders. Currently classified as a neurodevelopmental disorder in DSM-V, the symptoms need to be present early on (before the age of 12)^1^, and former classification systems DSM-IV^2^ and ICD-10^3^ required symptoms to be present even before age 7 and 6, respectively, i.e., about the time when children start school. ADHD is more common in boys than in girls with a ratio of about 4:1^4^.

ADHD is in many ways a heterogeneous disorder that has been associated particularly with cognitive and motivational deficits. According to the integrative view presented by Sonuga-Barke, multiple developmental pathways may lead on the background of person by environment interactions to ADHD: cognitive deficits related to executive functions (including attention, anticipation/preparation, cognitive control/response inhibition) may be related to dysfunctions in the mesocortical dopaminergic system and associated brain regions (including dorsolateral prefrontal cortex, but also posterior attention systems responsible for orienting and alerting), while impaired motivation (e.g. delay aversion) may be associated with dysfunctions in the mesolimbic dopaminergic system or ventral-striatal hyporesponsiveness to reward^5, 6^.

Dysfunctions in neuronal networks associated with attentional processes and cognitive control were documented in a series of studies using event-related potentials (ERPs) with children usually being 8 years or older^7–10^. Reduced ERP components related to attentional orienting (Cue-P3), response preparation (contingent negative variation, CNV) and cognitive control during response inhibition (Nogo-P3) have repeatedly been reported. The Cue-P3 is linked to the posterior attention network mainly modulated by noradrenaline though processes triggering the Cue-P3 could also be under dopaminergic influence^11^. The subsequent Cue-CNV is a slow cortical potential with central topography reflecting resources allocated for response preparation. It is probably generated in the dorsal anterior cingulate, frontal cortex and midbrain dopaminergic nuclei, susceptible to dopaminergic modulation, and a lower CNV amplitude may reflect a persisting deficit in ADHD^11–13^. The Nogo-P3 with sources in the prefrontal cortex and the anterior cingulate cortex (ACC) may reflect inhibitory response control modulated by dopaminergic activity. The Nogo-P3 amplitude reduction is also considered a persistent deficit in ADHD^10, 11, 13^. Motivational effects, which can be assessed in the lab inter alia by contrasting task blocks with and without motivational contingencies, were at least partly found to be larger in children with ADHD at the performance and neuronal level^14, 15^.

Heritability estimates of more than 70% suggest a genetic component in ADHD^16^. However, only relatively small effects between individual risk alleles and ADHD suggest that environmental factors (and gene x environment interactions, respectively) also play a significant role^17^. Environmental effects on brain development and behaviour may alter gene activity (without changing the order of their DNA sequence) via epigenetic modifications (like DNA methylation). So, epigenetic studies could be of particular value to unravel the underpinnings of ADHD. However, it has to be considered that epigenetic changes can be tissue specific^18^. So, effects found in peripheral tissue (e.g., blood, buccal cells) may not reflect methylation patterns in the brain.

So far, only a few epigenetic studies have been conducted in children showing ADHD symptoms. Some of the studies e.g., refs 19–22 considered candidate genes and, at least for the dopamine receptor D4 (*DRD4*) and the serotonin transporter (*SLC6A4*) gene, significant associations between methylation and ADHD behaviour were obtained in more than one study.

Methylome-wide analyses were done by Walton *et al*.^23^ and Wilmot *et al*.^24^. Using the ARIES (Accessible Resource for Integrated Epigenomics Studies) sample comprising more than 800 children, Walton *et al*.^23^ investigated if DNA methylation at birth was associated with trajectories of ADHD symptoms at the age of 7–15 years. They obtained 13 probes (annotated inter alia to *PEX2*, *SKI* and *ZNF544*) that were associated with later ADHD trajectories applying FDR (false discovery rate) correction. Gene network analysis revealed a complex interconnected network related to peroxisomal and neurodevelopmental processes. However, none of the probes turned out to be significant when considering methylation at age 7.

Wilmot *et al*.^24^ did their methylome-wide analysis in a smaller sample of boys with ADHD and typically developing boys aged 7–12 years. Several probes on the *VIPR2* and *MYT1L* gene fulfilled their less stringent statistical threshold and enrichment analysis indicated pathways related to inflammatory processes and modulation of monoamine and cholinergic neurotransmission.

Summarizing the results, epigenetic studies in ADHD do not provide a congruent picture by now but indicate that methylation patterns may be associated with ADHD but need not to be stable in the course of development. The functional relevance of methylation variations regarding attentional and response control processes have not been considered yet.

### Aims of the present study

First, we investigated whether ERP markers and performance measures reflecting attention, cognitive control and motivational aspects already show differences in boys at early school age depending on their ADHD behaviour (dimensional approach). As mentioned above, this age range has been underrepresented in previous studies though, according to DSM-IV and ICD-10, diagnostic criteria have to be fulfilled at this age.

Second, we studied associations between DNA methylation variations and ADHD behaviour and whether these variations are of functional relevance. In this respect, we tested if methylation levels are correlated with ADHD behaviour and associated performance and ERP measures, respectively. As sample size was too small to expect methylome-wide statistical significance (p < 1e-7), we considered candidate genes thought to play a role in the aetiology of ADHD (e.g., ‘classic’ ADHD genes as dopaminergic and noradrenergic genes, genes encoding proteins involved in cell adhesion and migration) as reviewed by Banaschewski *et al*.^25^ and genes found in the methylome-wide analysis of Wilmot *et al*.^24^ for children in a comparable age range.

Following a dimensional perspective of ADHD (interpreting ADHD as the extreme end of a continuous distribution of behaviour(s)^26^), we considered a sample of about 80 boys participating in a longitudinal study on child development. The associations of dimensional ADHD behaviour ratings, functional (performance and brain electrical activity) measures and DNA methylation were assessed cross-sectional.

## Results

### Go/nogo task – Performance and ERP measures

Results of the ANCOVAs (including the within-subject factor INCENTIVES and the dimensional covariate ADHD) are summarized in Table 1. For impulsivity errors, the factor INCENTIVES was found to be significant (medium effects size; F(1,80) = 11.5, p = 0.001; part. η^2^ = 0.13) indicating less impulsivity errors in blocks with motivational incentives (compared to blocks without motivational incentives).

**Table 1:** Motivational go/nogo task - results of the ANCOVA analyses (main and interaction effects) for the performance measures and ERP amplitudes [covariate ADHD (FBB-ADHS total score), within-subject factors INCENTIVES and ELECTRODE (only for the Nogo-P3)]. Abbreviations: CNV: contingent negative variation.

Measures	ANCOVA
Hits (N)	INCENTIVES: F(1,80) = 1.20, n.s.
	ADHD × INCENTIVES: F(1,80) = 0.35, n.s.
	ADHD: F(1,80) = 0.23, n.s.
Impulsivity errors (N + 1, log)	INCENTIVES: F(1,80) = 11.5, p = 0.001, part. η^2^ = 0.13
	ADHD × INCENTIVES: F(1,80) = 0.80, n.s.
	ADHD: F(1,80) = 2.34, n.s.
Reaction time (median, ms)	INCENTIVES: F(1,80) = 0.90, n.s.
	ADHD × INCENTIVES: F(1,80) = 2.96, n.s.
	ADHD: F(1,80) = 0.04, n.s.
Reaction time variability (ms)	INCENTIVES: F(1,80) = 0.11, n.s.
	**ADHD x INCENTIVES: F(1,80) = 5.69, p = 0.019, part. η** ^**2**^ ** = 0.07**
	ADHD: F(1,80) = 1.22, n.s.
Cue-P3 (Pz, µV)	INCENTIVES: F(1,80) = 0.27, n.s.
	ADHD × INCENTIVES: F(1,80) = 0.11, n.s.
	**ADHD: F(1,80) = 5.61, p = 0.020, part. η** ^**2**^ ** =0 .07**
CNV (Pz, µV)	INCENTIVES: F(1,80) = 5.31, n.s.
	ADHD × INCENTIVES: F(1,80) = 0.01, n.s.
	ADHD: F(1,80) = 1.45, n.s.
Go-P3 (Pz, µV)	INCENTIVES: F(1,80) = 1.20, n.s.
	ADHD × INCENTIVES: F(1,80) = 0.63, n.s.
	ADHD: F(1,80) = 0.61, n.s.
Nogo-P3 (CPz and Pz, µV)	INCENTIVES: F(1,80) = 0.01, n.s.;
	ELECTRODE: F(1,80) = 11.7, p = 0.001, part. η^2^ =0.13
	INCENTIVES × ELECTRODE: F(1,80) = 1.81, n.s.
	ADHD × INCENTIVES: F(1,80) = 0.19, n.s.
	ADHD × ELECTRODE: F(1,80) = 1.63, n.s.
	ADHD × INCENTIVES x ELECTRODE: F(1,80) = 0.06, n.s.
	**ADHD: F(1,80) = 5.60, p = 0.020, part. η** ^**2**^ ** = 0.07**

Regarding reaction time variability, a significant ADHD × INCENTIVES interaction effect (medium effect size; F(1,80) = 5.69, p = 0.019, part. η^2^ = 0.07) was obtained due to a larger influence of motivational contingencies with increasing FBB-ADHS total score. For the other performance parameters, no significant effects related to ADHD could be found (F(1,80) ≤ 2.96, p ≥ 0.089).

**Figure 1:**
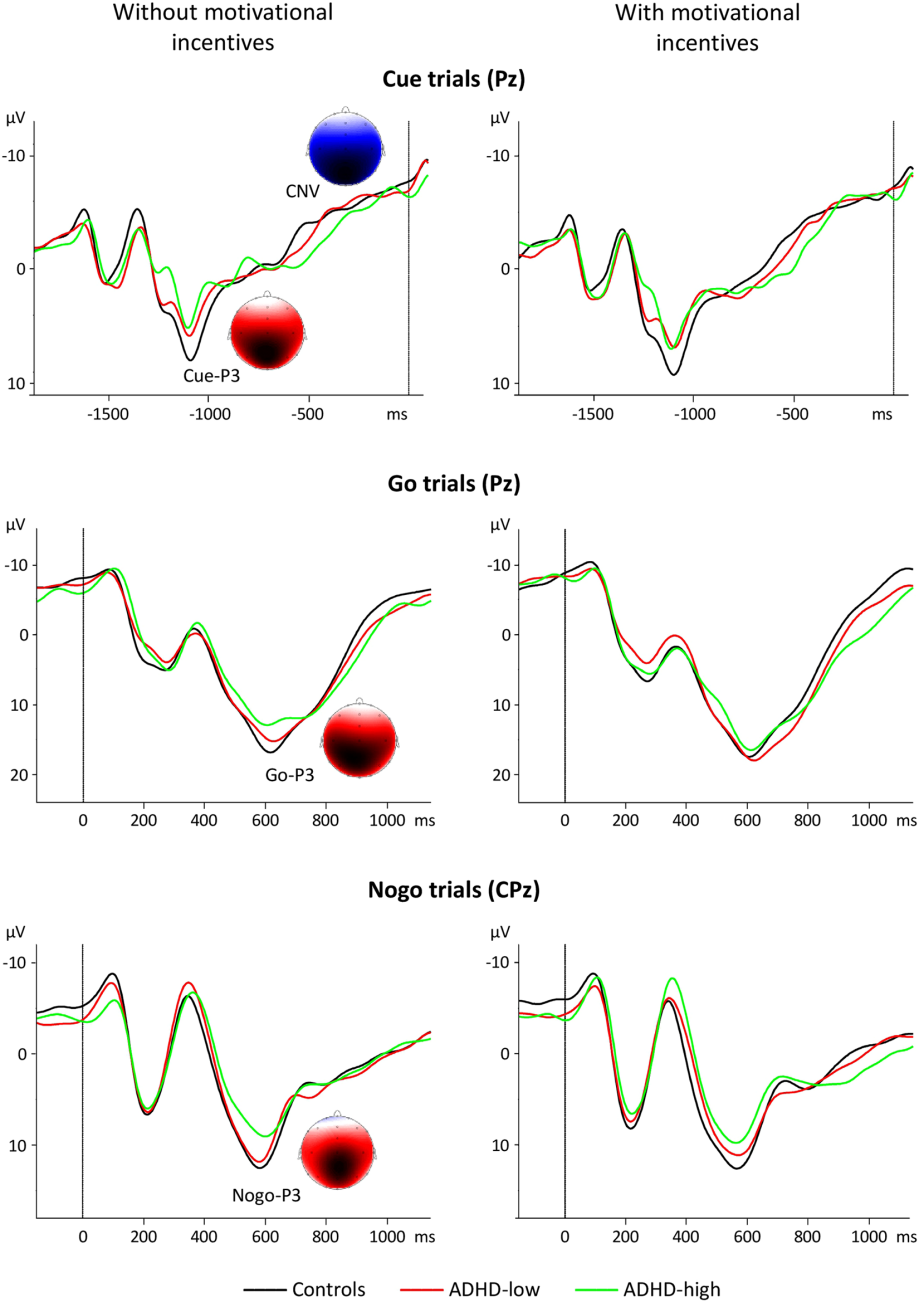
Grand average event-related potentials for boys with FBB-ADHS total scores ≤0.5 (controls; black curves), boys with FBB-ADHS total scores >0.5 and ≤1 (ADHD-low; red curves) and boys with FBB-ADHS total scores >1 (ADHD-high; green curves). Top: ERPs following cue stimuli (electrode Pz), middle: ERPs following go stimuli (electrode Pz), bottom: ERPs following nogo stimuli (electrode CPz). Signals obtained for the without-incentives (resp. with-incentives) condition are shown on the left (resp. right) side. Time point 0 ms refers to the onset of the S2 stimulus. Topographies of Cue-P3, CNV, Go-P3 and Nogo-P3 in the control group for the without-incentives condition are also depicted. Blue and red colours indicate negative and positive amplitude values, respectively.

For Cue-P3 and Nogo-P3, significant ADHD effects could be revealed (medium effect sizes; F(1,80) ≥5.60, p = 0.020, part. η^2^ = 0.07) indicating smaller amplitudes with increasing ADHD score. In Fig. 1, grand average ERPs for cue trials, go trials and nogo trials are depicted. Reduced Cue-P3 and Nogo-P3 amplitudes in boys with the highest ADHD scores (not depending on the INCENTIVES condition) are evident. For CNV and Go-P3 amplitudes, no significant effects regarding ADHD or ADHD × INCENTIVES interactions were obtained (F(1,80) ≤ 1.45, p ≥ 0.232). CNV amplitudes were higher in blocks with motivational incentives compared to blocks without incentives (as indicated by a significant INCENTIVES effect; F(1,80) = 5.31, p = 0.024, part. η^2^ = 0.06).

Comparing children with FBB-ADHS scores in the normal range ( ≤0.5, Controls, N = 40) and children with FBB-ADHS scores in the clinical range (>1, ADHD-high, N = 13) regarding the difference in reaction time variability between blocks with incentives and blocks without incentives, the Cue-P3 (at electrode Pz) and the Nogo-P3 (at electrode CPz) revealed significant effects for all three measures (post-hoc t-tests: |t(51)| ≥ 2.29, p ≤ 0.026; see also Table S1).

These three functional parameters were considered in the subsequent methylation analysis.

### Methylation results

DNA methylation data of 67 (of the 82) boys could be included in the analysis. Data from non-Caucasian children (N = 2) were not considered. Four children had to be excluded due to errors concerning sample collection and, in 9 children, DNA sample failed quality control.

Associations between probes, Nogo-P3 and ADHD behaviour fulfilling our statistical criteria were obtained for the *COMT*, *ANKK1*, *BDNF* and *NGFR* genes, i.e., genes related to the dopaminergic and neurotrophic system, respectively (see Table 2 and also Table S2). In post-hoc t-tests (comparing children with FBB-ADHS scores ≤ 0.5 and children with FBB-ADHS scores >1), these four CpGs were also found to be significant (|t(36–40)| ≥ 2.08, p ≤ 0.0436; see also Table S3). Regressions analyses with prenatal risk factors as covariates confirmed the results for these CpGs. Excluding those four children receiving methylphenidate revealed partly smaller effects. The association between the CpG linked to *BDNF* (cg11806762) and the FBB-ADHS total score was no longer significant (for details see Table S4).

**Table 2:** Results of the DNA methylation analysis.

Gene	CpG site	FBB-ADHS total score			dRTVar			Cue-P3			Nogo-P3		
		correlation	p-value	FDR	correlation	p-value	FDR	correlation	p-value	FDR	correlation	p-value	FDR
*COMT* − 29 CpGs	cg09926649	−0.424	0.0003	0.010									
	**cg08289189**	**0.395**	**0.0014**	**0.020**							**−0.361**	**0.0037**	**0.036**
	cg16834011										−0.375	0.0018	0.034
	cg07019740				0.250	0.0450		0.252	0.0429		0.371	0.0023	0.034
	cg23268677										0.313	0.0105	0.076
*ANKK1* − 10 CpGs	cg02682525	0.443	0.0002	0.002									
	cg16158779	0.403	0.0007	0.004									
	**cg15946653**	**0.386**	**0.0012**	**0.004**							**−0.315**	**0.0095**	**0.095**
	cg20667575	0.382	0.0014	0.004									
	cg16405454	0.305	0.0122	0.024									
*TPH2* − 19 CpGs	**cg14791008**	**0.426**	**0.0004**	**0.008**	**−0.364**	**0.0028**	**0.050**						
	cg18701449				−0.337	0.0053	0.050						
*BDNF* − 50 CpGs	**cg11806762**	**−0.263**	**0.0317**								**0.408**	**0.0006**	**0.031**
	cg11241206	0.327	0.0068										
	cg25457956										−0.301	0.0135	
*NGFR* − 24 CpGs	cg25226226	0.381	0.0015	0.027									
	cg17369032	0.367	0.0022	0.027									
	cg01438403	0.341	0.0048	0.035				−0.275	0.0241				
	**cg04613258**	**0.333**	**0.0058**	**0.035**							**−0.304**	**0.0124**	
*DPP10* − 45 CpGs	cg22670147							−0.390	0.0011	0.035			
	**cg19651219**	**0.320**	**0.0083**					**−0.380**	**0.0015**	**0.035**			
	cg24654266							−0.353	0.0034	0.051			
	cg21322022							−0.322	0.0080	0.090			
	cg00089091										0.309	0.0110	
	cg19211931							−0.307	0.0116				

Those genes fulfilling the combined statistical thresholds are presented. All probes linked to these genes with at least one correlation of medium effect are listed. Those CpGs fulfilling our statistical criteria (for details see text) are printed in bold. Only FDRs < 0.1 are listed. Abbreviations: FBB-ADHS (German ADHD rating scale); FDR = false discovery rate; dRTVar = difference of reaction time variability between blocks with and without motivational incentives.

**Table 3:** Sample characteristics for the 82 boys selected from the FRANCES sample.

Parameters	M ± SD/N (%)
Age (years)	7.54 ± 0.57
IQ^44^	104.5 ± 9.8
Socioeconomic status	11.4 ± 2.0
ADHD rating scale - total score^45^	0.64 ± 0.48
Prenatal risk factors:	
Maternal smoking	12 (14.6%)
Alcohol exposure	18 (22.0%)
Maternal depressive symptomatology	12 (14.6%)

The socioeconomic status (sum index) was calculated on the basis of maternal and paternal secondary education level and family income (theoretical range: 3–14; higher values indicating higher status). Presence of prenatal risk factors maternal smoking (self-report: cutoff: 1 cigarette/day), prenatal alcohol exposure (meconium ethyl glucuronide; cutoff: 10 ng/g) and prenatal maternal depressive symptomatology (Edinburgh Postnatal Depression Scale, EPDS^50^; cutoff: 10) was not considered as an exclusion criterion.

Computing linear regressions (backward elimination) starting with those four CpG sites fulfilling our statistical criteria as predictor variables, three CpGs remained in the model for the Nogo-P3 (*COMT* - cg08289189: p = 0.002; *ANKK1* - cg15946653: p = 0.036; *BDNF* - cg11806762: p = 0.0002) explaining about 34% of the variance (adjusted R^2^ = 0.343; F(3,59) = 11.8, p = 3.69e-06) and in the model regarding the FBB-ADHS total score (*COMT* - cg08289189: p = 0.001; *ANKK1* - cg15946653: p = 0.003; *BDNF* - cg11806762: p = 0.023) explaining ca. 31% of the variance (adjusted R^2^ = 0.308; F(3,59) = 10.2, p = 1.69e-05), while the CpG linked to *NGFR* (cg04613258) fell short of significance.

CpG site cg19651219 (linked to the *DPP10* gene) was associated with the Cue-P3 and ADHD behaviour.

**Figure 2:**
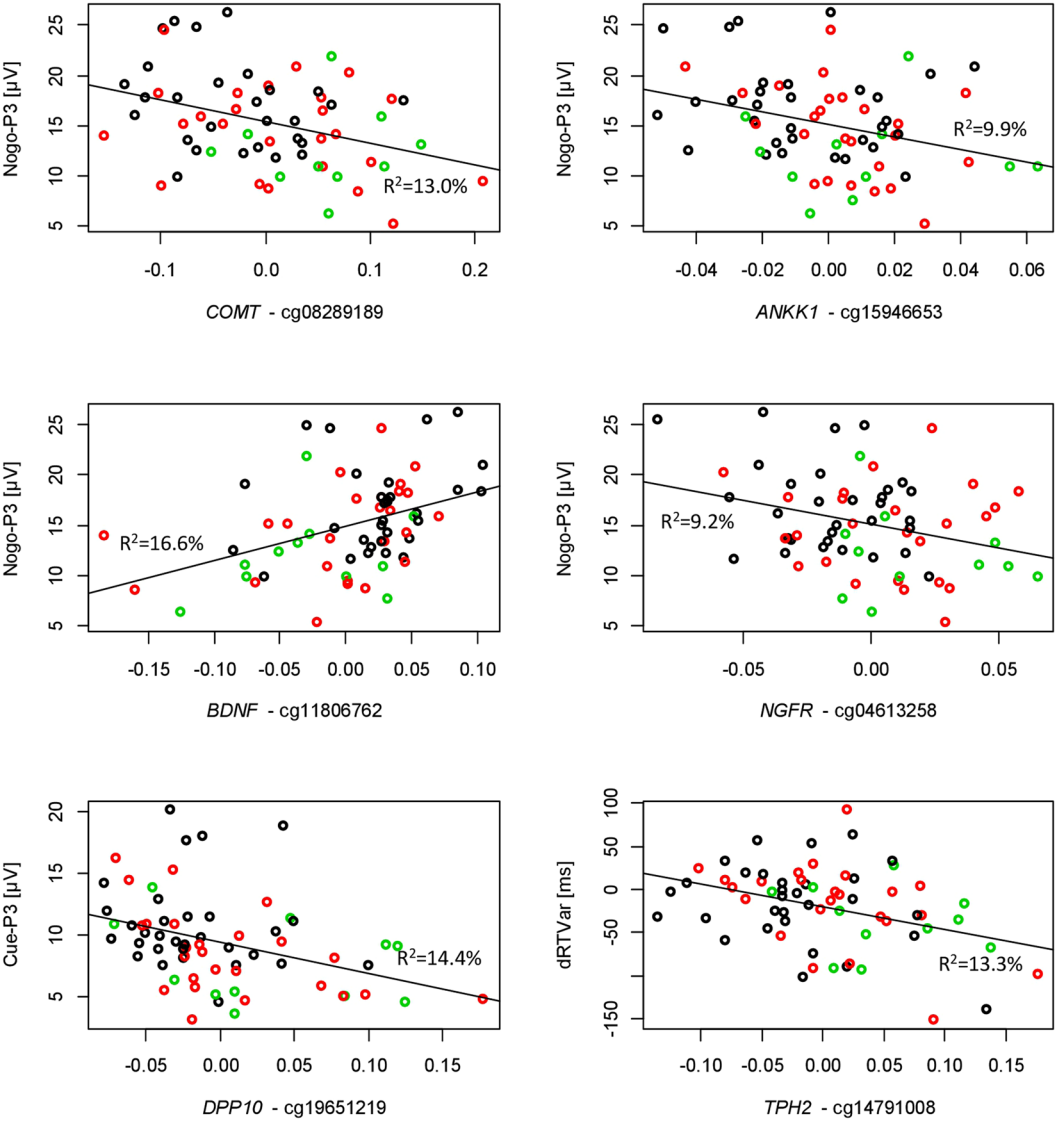
Scatter plots (with regression lines) showing associations between DNA methylation and functional measures (boys with FBB-ADHS total scores ≤0.5, controls: black circles; boys with FBB-ADHS total scores >0.5 and ≤1, ADHD-low: red circles; boys with FBB-ADHS total scores >1, ADHD-high: green circles). 5 of the 6 CpGs were hypermethylated in the groups with higher ADHD scores. It has to be noted that residuals are depicted which are centered at 0. dRTVar = difference of reaction time variability between blocks with and without motivational incentives.

For *TPH2*, probe cg14791008 correlated with the incentive-dependent reaction time variability effect and ADHD behaviour (see also Fig. 2 and Fig. S1).

## Discussion

In order to enhance our understanding of ADHD, we investigated attentional, cognitive control and motivational processes in boys at early school age and associations with DNA methylation variations. To our knowledge, it is the first study linking DNA methylation and functional aspects related to ADHD. Using a non-clinical sample, boys with a higher ADHD score showed smaller Cue-P3 amplitude (reflecting attentional orienting) and a reduced Nogo-P3 (reflecting inhibitory response control) as well as a larger motivational influence on reaction time variability in a go/nogo task. ERP effects were of medium-to-large effect sizes. For several candidate genes, associations of CpGs sites with those functional aspects and with ADHD behaviour of at least medium size were obtained.

### Attention, cognitive control and motivation in ADHD

Reduced Cue-P3 and Nogo-P3 components have been found in older children and adolescents with ADHD^7^ as well as in adult patients with ADHD^10^ and, thus, may be considered as a robust finding in ADHD. Spronk *et al*.^27^ also investigated relatively young (5–7 years old) children and, as in our study, children did not have a clinical diagnosis of ADHD. They also obtained a reduced Cue-P3 and at least a tendency for a reduced Nogo-P3 in their small sample. So, it may be concluded that deficient attentional orienting and inhibitory response control characterize (at least) boys with ADHD from mid-childhood into adulthood. Findings from our present study also indicate that these deficits are not related to motivational issues.

For the CNV (reflecting cognition preparation), the major part of the studies reported a reduced amplitude at central electrodes in patients with ADHD though findings are not as consistent as for the Cue-P3 and the Nogo-P3. Spronk *et al*.^27^ even observed a larger CNV at occipital sites in their 5–7 years old group of children with ADHD symptoms. So, due to developmental effects, the central CNV may be considered as a neurophysiological marker for ADHD starting in late childhood.

A meta-analysis^28^ revealed a large effect size for increased reaction time variability in children and adolescents with ADHD but this could not be found in our data, probably due to the character of the task (using a S1–S2 paradigm). However, a larger decrease of reaction time variability in the blocks with motivational contingencies in the group of children with a higher ADHD score may be seen in line with the notion of motivational processes as a pathway to ADHD^5^. The number of impulsivity errors was not significantly higher with increasing ADHD score, which is in line with other studies using a cued go/nogo task e.g., refs 9, 10. The inhibition deficit in ADHD becomes evident at the performance level only in more challenging tasks (e.g., the stop task)^7^.

### DNA methylation effects

The genes, for which our data showed associations of CpGs sites with functional aspects and ADHD behaviour fulfilling our statistical criteria (*COMT*, *ANKK1*, *BDNF*, *NGFR*, *DPP10*, *TPH2*), have not been considered in the candidate-gene methylation studies in ADHD e.g., refs 19–22 and have not been reported to be differentially methylated in the methylome-wide studies^23, 24^.

Findings did not change when including prenatal maternal smoking, prenatal alcohol exposure and prenatal depressive symptomatology as covariates indicating that methylation effects were not induced by these prenatal risk factors. Effects were partly smaller when excluding children receiving methlpenidate. However, it has to be considered that children on medication are typically more severe cases. So, including them probably increases representativeness^24^. We could not confirm the findings of the case-control-study of Wilmot *et al*.^24^ regarding *VIPR2* and *MYT1L* in our dimensional analysis.

Associations of probes linked to genes of the dopaminergic system (*COMT*, *ANKK1*) with the Nogo-P3 and ADHD behaviour further support the notion that the dopaminergic system is affected in ADHD. COMT (catechol-o-methyltransferase) is critical for monoamine signaling at the site of cortical synapses. Interestingly, in a study related to malnutrition, an association of a CpG site with attention (ADHD score) in adults was reported^29^ though it was a different CpG site than the CpG sites we found in our study.

The *ANKK1* (ankyrin repeat and kinase domain containing 1) gene was originally associated with the *DRD2* gene. Due to its close proximity to *DRD2*, *ANKK1* is assumed to regulate *DRD2*. It may also play a role in the development of alcohol dependence. In a recent methylation study related to maltreatment, children with early onset maltreatment had higher methylation values at *ANKK1* indicating an increased risk for adverse mental health outcomes^30^. These effects may complement the association of Nogo-P3 and impulsive behaviour in a cued continuous performance test with a DAT1 haplotype^11^ but further research is needed to link developmental with genetic and epigenetic effects to actual phenotypes.

The *BDNF* (brain derived neuroptrophic factor) gene and the *NGFR* (nerve growth factor receptor) gene belong to the neurotrophin family. They are involved in the development, plasticity and survival of dopaminergic and serotonergic neurons and may play an important role regarding learning and memory but also cognitive functions. Up to now, methylation studies related to *BNDF* have mainly been conducted in patients with depression, bipolar disorder and schizophrenia^31–33^.

Regarding the Cue-P3, only an association with CpG sites in the *DPP10* was detected. The *DPP10* gene encodes dipeptyl peptidase 10, which binds to specific voltage-gated potassium channels and alters their expression. Potassium channel function can affect dopaminergic tone and may thus be involved in the development of neuropsychiatric disorders like schizophrenia, autism and ADHD^25, 34^. As the Cue-P3 is linked to the posterior attention network mainly modulated by noradrenaline^8, 35^, we would have expected associations with corresponding CpG sites. Applying less stringent criteria (see Table S2) revealed at least one association of medium effect size (FDR of ca. 0.1) with a CpG site linked to the noradrenaline transporter (*NET1*/*SLC6A2*) gene. Moreover, in 3 of the 17 CpG sites linked to the *DRD4* gene, associations with the Cue-P3 were nominally significant (p < 0.05) which can be seen in line with the findings of Albrecht *et al*. (2014) that processes triggering the Cue-P3 response may be influenced by *DRD4* 7 R polymorphisms (also mildly associated with ADHD) or the study by Gizer & Waldman^36^ reporting *DRD4* 7 R related to inattention.

In our data, we obtained an association between CpG sites in the *TPH2* gene and the influence of motivational incentives on reaction time variability. *TPH2* encodes tryptophan hydroxylase which is the rate-limiting enzyme in the production of serotonin. We are not aware of other studies considering DNA methylation in the *TPH2* gene related to reward aspects. Guillemin *et al*.^37^ found that the promotor of *TPH2* is differentially methylated in adults already showing aggressive behaviour in childhood. Interestingly, Neufang *et al*.^38^ reported that the *TPH2* genotype (GG homozygotes vs T allele carriers) modulated the response to monetary rewards in the nucleus accumbens which could provide a link between *TPH2*, reward-related processes and impulsive behaviour.

In summary, reflecting the literature may indicate that DNA methylation variations in those genes, for which we obtained significant findings, are involved in the development of neuropsychiatric disorders like ADHD. However, as only limited data are available by now, it does not allow to support our findings directly.

### Limitations and open questions

ADHD-related behaviour was only assessed via maternal ratings which may be biased. On the other hand, ratings by teachers, who divide their attention over 20–30 children in a class and observe a child only for a limited period during the day, might be more prone to measurement error^39^.

Sample size was relatively small in our study, and a confirmation sample was not available. So, replication in a larger sample is urgently needed. Larger sample will also allow methylome-wide analysis (as has been done for example by Walton *et al*.^23^) and to study to what extent findings are comparable or different for boys and girls. Generally, it has to be noted that findings obtained in the methylation studies in ADHD do not provide a converging picture. Differences in age ranges considered (methylation at birth vs. early or later childhood), sample composition (case-control vs. population-based/non-clinical) and methodological issues regarding methylation analysis may account for the non-congruent picture.

If a more conservative approach to correct for multiple testing was used, most of the ERP and epigenetic findings would no longer be significant. However, since performance and ERP measures are considered as independent (reflecting different aspects of attention and cognitive control), it is common practice in ERP studies not to correct for multiple testing^8–11, 13^. Our statistical criteria for the methylation data may be seen as a compromise between limited sample size and reducing the probability of false positive results.

It remains open to what extent methylation variations in the identified CpG sites exert functional effects on gene expression and activity as no RNA was available. Moreover, genetic influences and further environmental factors (as well as their interactions) need to be considered for better understanding biological vulnerability and ADHD.

Lastly, epigenetic changes can be tissue specific so that methylation patterns in the brain may be different than in peripheral tissue (and vice versa). However, at least for *COMT* and *BDNF*, there are some hints for (strong) correlations between methylation variations in peripheral and brain tissue^40, 41^ but more research is needed before the associations found in our current study can be integrated into functional pathways from genes, environment and epigenetic processes towards brain functions and mental disorders.

## Conclusions

The following conclusions may be drawn from our study:

1. Diminished attentional orienting, reduced inhibitory response control and larger motivational performance effects appear to characterize boys with ADHD symptoms already at early school age.
2. DNA methylation variations may be associated with functional deficits in ADHD. Particularly, the dopaminergic system and the neurotrophic system could contribute to the response control deficits in ADHD.
3. The associations between methylation and brain functions may, at least partly, not be specific to ADHD but may also occur in other disorders as genes like *COMT*, *BDNF* and *DPP10* are considered as neuropsychiatric risk genes.

Despite the limitations of our study, we expect that future studies targeting associations between DNA methylation, brain functions (resp. associated circuits) and behaviour with larger samples longitudinally will further enhance our understanding of ADHD. Using larger samples will allow to conduct methylome-wide analysis and to address the heterogeneity of the disorder.

## Materials and Methods

### Participants and procedures

82 boys (aged 6 to 8 years) were included in the present investigation. They were selected from the FRANCES (Franconian Cognition and Emotion Studies) cohort. FRANCES, a follow-up study of a prospective longitudinal study in a region located in the southern part of Germany (FRAMES, Franconian Maternal Health Evaluation Study^42^), aims at investigating the effects of prenatal risk factors on child development at early school age^43^.

In FRANCES, 618 women who had participated in FRAMES were contacted and a total of 245 children (127 boys) agreed to participate. 215 children (113 boys) took part in the EEG lab session. Only those boys/families (N = 91), who responded to an invitation letter (but were not actively contacted in a later phase of recruiting due to prenatal risk factors like alcohol exposure, prenatal depression or smoking), were included here to prevent an overrepresentation of prenatal risk factors and to achieve a more representative sample. Children did not use psychotropic medication except for four boys taking methylphenidate. These four boys were at least drug-free for 24 hours before the assessments. Children with an IQ less than 75 (N = 1, estimated with the Intelligence and Development Scales, IDS^44^) were excluded as well as children with insufficient comprehension of the go/nogo task or poor EEG data quality (N = 8). Sample characteristics are summarised in Table 3.

ADHD-related behaviour was assessed via the German ADHD rating scale (FBB-ADHS)^45^ rated by the mothers. This questionnaire comprises 20 items (9 items related to inattention, 7 items related to hyperactivity and 4 items related to impulsivity). Each item is rated from 0 (‘not at all’) to 3 (‘notably’). The total score represents the mean value across all 20 items.

Recording of event-related potentials during a motivational go/nogo task and DNA collection took place at the same day.

The study was approved by the Ethics Committee of the University Hospital of Erlangen and conducted in accordance to the declaration of Helsinki. Parents and children received written information and provided informed consent.

### Cued go/nogo task

**Figure 3:**
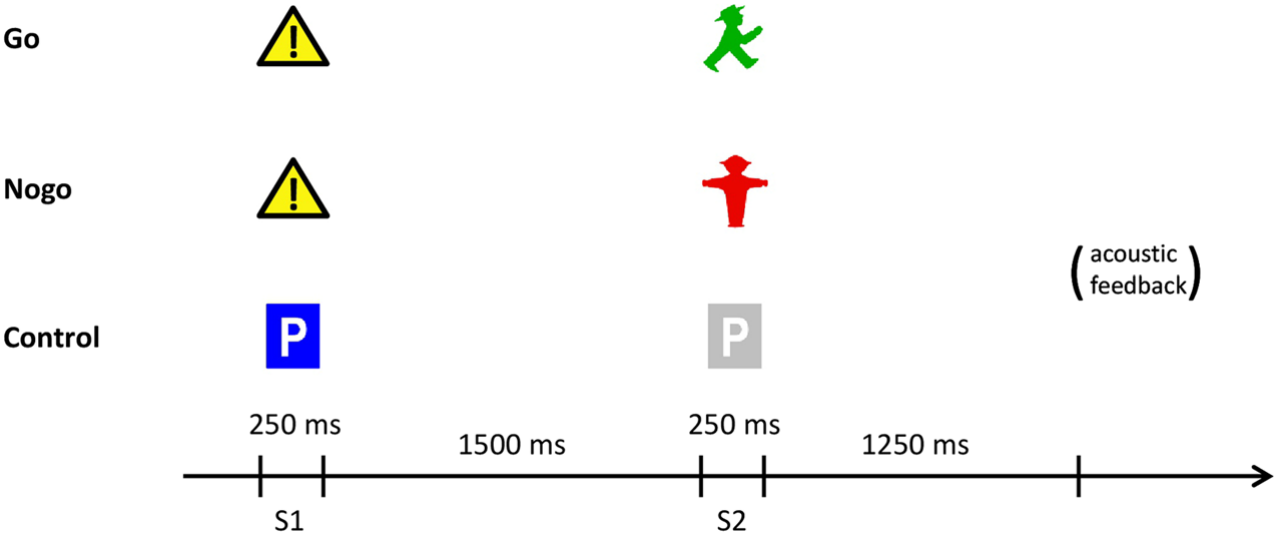
Schematic illustration of the cued go/nogo task (S1–S2 paradigm). Traffic sign icons were used as stimuli. All trial types (go, nogo and control) occurred with equal probability. In block 2 and block 3 of the task, monetary incentives were used. Money won or lost in a trial was indicated by corresponding acoustic feedback. For details see text.

The task (for schematic illustration see Fig. 3) consisted of four blocks comprising 36 trials each. Each trial started with the presentation of a cue stimulus of 250 ms duration, which was followed by a test stimulus of 250 ms duration (S1–S2 paradigm). The interval between S1 and S2 (stimulus onset asynchrony) was 1750 ms, the intertrial interval (S1-S1) was set randomly to 3500 ± 500 ms.

Three different kinds of trials occurred with equal probability:

- go trials: S1 - a danger traffic sign; S2 - the green figure of pedestrian traffic lights
- nogo trials: S1 - a danger traffic sign; S2 - the red figure of pedestrian traffic lights
- control trials: S1 - a blue parking sign; S2 - a greyed parking sign.

In the second and third block of the task (ABBA design), a monetary reward (10 cent per trial) was given for fast responses in go trials to increase motivation. The reaction time threshold for receiving rewards was dynamically adjusted to the 75th percentile of reaction times in go trials of the previous block applying a tracking algorithm^9^. In the case of a wrong reaction (reaction to a nogo trial or no reaction to a go stimulus within 1500 ms), the same amount of money was subtracted. The participants received acoustic feedback for fast, correct responses and incorrect responses.

The number of hits, impulsivity errors, the median reaction time as well as reaction time variability were determined and considered in the analysis.

The task was implemented using Presentation (Neurobehavioral Systems, Berkeley, CA,USA).

### ERPs - recording and analysis

During the cued go/nogo task, EEG activity was recorded from 25 electrodes (10/20-system plus additional midline electrodes Fpz, FCz (recording reference), CPz, Oz and mastoid electrodes TP9 and TP10; ground electrode: CP2). Electrode caps with sintered Ag/AgCl electrodes (Easycap, Herrsching, Germany) were used. Impedances had to be below 20 kΩ. Raw data were recorded with a Brainproducts system (standard Brainamp amplifier and Vision Recorder software; Brainproducts, Gilching, Germany) at a sampling rate of 500 Hz (filter bandwidth: 0.016–120 Hz).

Preprocessing steps comprised downsampling to 250 Hz, filtering (bandpass: 0.5–20 Hz; 24 db/Oct Butterworth filters and 50 Hz notch filter), correction of artefacts induced by blinks and eye movements^46^ and re-referencing to linked mastoids. Amplitudes exceeding ± 150 μV were interpreted as artefact and corresponding segments as well as trials with performance issues (errors or responses faster than 200 ms or slower than 1500 ms after the S2 stimulus) were excluded from further ERP analyses.

The following segments were built (related to the S2 stimulus): cue segments ranging from −1850 ms to 100 ms and go and nogo segments lasting from −150 ms to 1150 ms. After averaging, ERP components were measured at the electrode(s) with highest amplitude: CNV (mean amplitude from −500 ms to 0 ms; Pz), Cue-P3 (−1300 ms to −1000 ms; Pz) and Go-P3 (maximum amplitude within 300 ms to 700 ms; Pz) and Nogo-P3 (maximum amplitude within 300 ms to 700 ms; CPz and also Pz).

### DNA extraction and methylation analysis

For our study, DNA was extracted from buccal cells and analysed with the Infinium Human Methylation 450K BeadChip (Illumina, San Diego, CA, USA) by the Helmholtz-Zentrum München (Germany)^47^. Buccal cells were collected from participants using buccal swabs (OmniSwab (Wb100035, Whatman, Maidstone, UK) and stored at 4 °C. Subsequently, DNA was extracted using DNeasy Blood & Tissue Kit (Qiagen, Germany, Cat. No. 69506) according to the manufacturer’s recommendation. A total of 500 ng purified DNA was used for further methylation analysis.

Preprocessing of the methylation data was based on the pipeline of Lehne *et al*.^48^ and implemented in R (v. 3.3.1) using the minfi and wateRmelon packages. It was performed on all children (boys and girls) of the complete FRANCES sample with DNA methylation data of sufficient quality available (N = 174). Illumina Background correction was applied to the raw intensity values. Probes with a detection p-value ≥ 0.001, containing SNPs or located on gender chromosomes were removed as were samples with a call rate < 97%. Raw intensity values were normalized using quantile normalization and subsequently converted to beta values, as the proportion of DNA methylated at a single CpG site (values from 0 to 1). Probes with mean values below 0.01 and above 0.99, respectively, were not considered further. Values four standard deviations above or below the mean value were considered as outliers and excluded from further analysis.

Control probe adjustment was used to reduce technical bias, which appears to be superior to using experimental factors like array number or position on array^48^. In this regression-based approach, a principal component analysis (PCA) of the control probes is computed first to reduce multicollinearity as control probes are highly correlated. Besides the 22 PCA factors (explaining 95% of the variance), sex and age were included as predictors in a linear regression model (predicting beta values). As shown in Fig. S2, technical bias (plate and chip effects) was effectively reduced by this approach.

A second PCA was performed on the residuals (obtained from the first regression analysis) to account for further variance (biological factors, global covariation). The first two factors of this second PCA were included as additional predictors (besides sex and age and the 22 factors of the first PCA) in a second (final) regression model (predicting beta values). The resulting residuals (reflecting adjusted methylation values) were used for further analysis.

### Candidate genes

Selection of candidate genes was primarily based on a review by Banaschewski *et al*.^25^. We considered CpG methylation in the following genes:

- genes involved in the dopaminergic system: dopamine receptor genes (*DRD1*, *DRD2*, *DRD3*, *DRD4*, *DRD5*), *DAT1*/*SLC6A3*, *COMT*, *ANKK1*, *DDC*, *DBH*
- genes involved in the noradrenergic system: *NET1*/*SLC6A2*, *ADRA2A*, *ADRA2C*, *ADRA1A*, *ADRA1B*, *ADRB1*, *ADRB2*
- genes involved in the serotonergic system: *5-HTT*/*SLC6A4*, *HTR1B*, *HTR2A*, *TPH2*
- genes involved in the neurotrophic system: *BDNF*, *NGF*, *NGFR*, *NTF3*, *NTF4*, *CNTF*, *CNTFR*, *GDNF*, *NTRK1*, *NTRK2*, *NTRK3*
- genes encoding proteins involved in cell adhesion and migration: *CDH13*, *GFOD1*, *MTA3*, *SPATA13*, *UNC5B*, *ASTN2*, *CSMD2*, *ITGAE*, *ITGA11*, *CDH23*, *GPC6*, *CTNNA2*, *NAV2*
- genes encoding proteins related to potassium-mediated signaling: *KCNIP4*, *KCNIP1*, *DPP10*, *FHIT*, *KCNC1*.
- other candidate genes: *SNAP25*, *CHRNA4*, *SLC9A9*, *CNR1*, *NOS1*, glutamate (NMDA) receptor genes.

Additionally, we analysed CpGs linked to *VIPR2* and *MYT1L*, i.e., those genes identified in a methylome-wide study on ADHD in boys aged 7–12^24^.

We studied all CpGs linked to those genes as defined in the HumanMethylation450K Manifest File (v.1.2) except probes containing SNPs and virtually showing no variance (as described above). In total, 60 genes (2031 CpG sites, respectively) were included in the analysis (see Table S5).

### Statistical analysis

All performance measures and ERP measures (CNV, Cue-P3, Go-P3 and Nogo-P3) were subjected to ANCOVAs containing the within-subject factor INCENTIVES (task blocks without incentives, i.e., blocks 1 and 4; task blocks with incentives, i.e., blocks 2 and 3) and the covariate ADHD (FBB-ADHS total score). Main and interaction effects were interpreted. For the Nogo-P3 amplitude, an additional factor ELECTRODE (CPz, Pz) was introduced to take a possible differential nogo-anteriorization effect into account.

For these analyses, IBM SPSS v.21.0 was used. Statistical significance was assumed if p < 0.05. Effect sizes (partial η^2^) were also considered with part. η^2^ > 0.01 indicating small effects, part. η^2^ > 0.06 reflecting medium effects, and part. η^2^ > 0.14 representing large effects^49^.

For post-hoc analysis and also for illustration purposes, we also applied a categorial approach and divided the boys into three groups according their FBB-ADHS total score: controls (≤0.5); ADHD-low: (>0.5/≤1); ADHD-high (>1); see Table S6. These thresholds typically are used in case-control studies on ADHD^9^.

Associations between methylation and behavioural (ADHD) level and methylation and functional level (performance measures, ERP components), respectively, were tested using Pearson’s correlation coefficients (r). Only functional measures that showed differential effects regarding ADHD behaviour were considered. As these measures were not correlated with age in the narrow age range of our sample, the factor age was not controlled for when testing those associations.

To reduce the probability of false positive effects, four criteria had to be fulfilled:

1. |r| ≥ 0.3 correlations of CpGs linked to a gene with the ADHD score and one of the functional measures (corresponding to a p-value of 0.0135 for 67 samples)
2. FDR < 0.05 for an association between a certain CpG and either the behavioural or the functional level (Benjamini-Hochberg procedure, controlling for the number of probes linked to this gene)
3. p < 0.05 correlation for that CpG with the other level
4. |r| ≥ 0.3 correlation of a second CpG of this gene with the level involved in criterion (2).

To test whether methylation findings were affected by prenatal risk factors, we additionally computed regression models for the CpGs fulfilling our statistical criteria with maternal smoking, alcohol exposure and presence/absence of maternal depressive symptomatology during pregnancy as covariates. As the 24 h-washout may be considered too short to exclude a medication effect on DNA methylation, we repeated the correlational analyses without the four participants receiving methylphenidate.

If a functional measure turned out to be associated with more than one probe fulfilling the above-mentioned criteria, we computed linear regression models for the functional measure and the FBB-ADHS total score considering these probes as predictor variables (stepwise backward elimination process; variables with p > 0.05 being removed). No other covariates were included in the analysis.

Statistical analyses related to the methylation data were conducted using R (v.3.3.1).

## Electronic supplementary material

Supplementary Material

Table S-2
