# Supplementary material for: Attention, cognitive control and motivation in ADHD: Linking event-related brain potentials and DNA methylation patterns in boys at early school age
Source: Sci Rep. 2017 Jun 19;7:3823. doi: 10.1038/s41598-017-03326-3 (PMC5476641; doi:10.1038/s41598-017-03326-3)
Supplement: Supplementary file 1 — Supplementary Material [file 41598_2017_3326_MOESM1_ESM.pdf]

Supplementary Material

Attention, cognitive control and motivation in ADHD: Linking event-related brain potentials and DNA methylation patterns in boys at early school age

Hartmut Heinrich, Juliane Grunitz, Valeska Stonawski, Stefan Frey, Simone Wahl, Björn Albrecht, Tamme W. Goecke, Matthias W. Beckmann, Johannes Kornhuber, Peter A. Fasching, Gunther H. Moll, Anna Eichler

Table S-1: Performance and event-related potential (ERP) measures for the three groups of boys classified along their FBB-ADHS total score

Table S-2: Results of the DNA methylation analysis (less stringent statistical criteria) -> separate Excel file

Table S-3: Mean DNA methylation (± SD) for the three groups of boys classified along their FBB-ADHS total score

Table S-4: Additional analyses taking prenatal risk factors (maternal smoking, alcohol exposure, maternal depressive symptomatology) and medication into account

Figure S-1: Scatter plots (with regression lines) showing associations between DNA methylation and FBB-ADHS total scores

Figure S-2: Principal component analysis (PCA) before and after control probe adjustment

Table S-5: Candidate genes and number of CpGs

Table S-6: Sample characteristics for three groups of boys classified along their FBB-ADHS total score

Table S-1: Performance and event-related potential (ERP) measures for the three groups of boys classified along their FBB-ADHS total score: Controls (≤ 0.5); ADHD-low: (> 0.5 / ≤1); ADHD-high (> 1). Mean values ± standard deviations for those measures being associated with the FBB-ADHS total score are presented. Comparing Controls vs. ADHD-high using t-tests revealed significant results for all measures. Abbreviations: w/o = without; dRTVar = difference of reaction time variability between blocks with and without motivational incentives.

	Controls (N = 40)		ADHD-low (N = 29)		ADHD-high (N = 13)	
	w/o incentives	incentives	w/o incentives	incentives	w/o incentives	incentives
Reaction time variability (ms)	117.3 ± 48.9	104.4 ± 34.4	114.8 ± 50.3	100.8 ± 32.2	150.4 ± 58.9	107.1 ± 38.4
Cue-P3 (Pz, µV)	10.3 ± 3.6	10.9 ± 3.6	8.7 ± 3.7	8.9 ± 4.0	7.4 ± 3.6	8.2 ± 3.4
Nogo-P3						
CPz (µV)	16.8 ± 5.8	16.8 ± 4.4	15.5 ± 6.1	15.2 ± 5.3	12.1 ± 3.6	12.3 ± 5.7
Pz (µV)	15.1 ± 5.9	15.6 ± 4.5	14.6 ± 5.8	14.5 ± 5.9	11.1 ± 4.5	12.3 ± 6.4

Performance and ERP measures	Comparisons of Controls and ADHD-high (t-tests)	
dRTVar (ms)	t(51) = -2.29	p = 0.026
Cue-P3 (Pz)	t(51) = 2.74	p = 0.008
Nogo-P3 (CPz)	t(51) = 3.21	p = 0.002

Table S-3: Mean DNA methylation ± SD (residuals of the preprocessing regression analysis) for the three groups of boys classified along their FBB-ADHS total score: Controls (≤ 0.5); ADHD-low: (> 0.5 / ≤1); ADHD-high (> 1). Only CpG sites are included that fulfilled our statistical criteria (inter alia significant assocations with the behavioural and functional level). Comparing Controls vs. ADHD-high using t-tests revealed significant results for all CpGs.

Gene – CpG	Controls (N=31)	ADHD-low (N=25)	ADHD-high (N=11)
COMT - cg08289189	-0.0295 ± 0.0652	0.0145 ± 0.0857	0.0566 ± 0.0611
ANKK1 - cg15946653	-0.0074 ± 0.0240	0.0047 ± 0.0195	0.0109 ± 0.0282
BDNF - cg11806762	0.0250 ± 0.0450	-0.0016 ± 0.0621	-0.0276 ± 0.0534
NGFR - cg04613258	-0.0149 ± 0.0251	0.0070 ± 0.0296	0.0183 ± 0.0287
DPP10 - cg19651219	-0.0176 ± 0.0412	0.0065 ± 0.0582	0.0328 ± 0.0698
TPH2 - cg14791008	-0.0221 ± 0.0608	0.0097 ± 0.0628	0.0507 ± 0.0570

Gene – CpG	Comparisons of Controls and ADHD-high (t-tests)	
COMT - cg08289189	t(36) = -3.64	p = 0.0008
ANKK1 - cg15946653	t(40) = -2.08	p = 0.0436
BDNF - cg11806762	t(40) = 3.17	p = 0.0029
NGFR - cg04613258	t(40) = -3.64	p = 0.0008
DPP10 - cg19651219	t(40) = -2.88	p = 0.0064
TPH2 - cg14791008	t(39) = -3.45	p = 0.0013

Table S-4: Additional analyses taking prenatal risk factors (maternal smoking, alcohol exposure, maternal depressive symptomatology) and medication into account. For the linear regression models, coefficients ± standard errors are presented.

Linear regression analyses - prenatal risk factors as covariates

Gene – CpG	Associations with	
	ADHD total score	Nogo-P3
COMT - cg08289189	0.0401 ± 0.0139, p = 0.0053	-0.0065 ± 0.0020, p = 0.0024
ANKK1 - cg15946653	0.0213 ± 0.0058, p = 0.0005	-0.0017 ± 0.0006, p = 0.0072
BDNF - cg11806762	-0.0287 ± 0.0134, p = 0.0361	0.0043 ± 0.0013, p = 0.0022
NGFR - cg04613258	0.0204 ± 0.0077, p = 0.0098	-0.0019 ± 0.0008, p = 0.0179
		Cue-P3
DPP10 - cg19651219	0.0401 ± 0.0139, p = 0.0053	-0.0057 ± 0.0018, p = 0.0019
		dRTVar
TPH2 - cg14791008	0.0550 ± 0.0156, p = 0.0008	-4.8e-04 ± 1.6e-04, p = 0.0045

Correlation analyses - excluding participants receiving methylphenidate (MPH, N = 4)

Gene - CpG	Associations with	
	ADHD total score	Nogo-P3
COMT - cg08289189	r = 0.365, p = 0.0045	r = -0.341, p = 0.0083
ANKK1 - cg15946653	r = 0.367, p = 0.0031	r = -0.306, p = 0.0147
BDNF - cg11806762	r = -0.202, p = 0.1125	r = 0.376, p = 0.0024
NGFR - cg04613258	r = 0.290, p = 0.0213	r = -0.28134, p = 0.0256
		Cue-P3
DPP10 - cg19651219	r = 0.367, p = 0.0031	r = -0.361, p = 0.0037
		dRTVar
TPH2 - cg14791008	r = 0.405, p = 0.0012	r = -0.378, p = 0.0027

Figure S-1: Scatter plots (with regression lines) showing associations between DNA methylation and the FBB-ADHS total score. 5 of the 6 CpGs were hypermethylated in boys with higher ADHD scores. It has to be noted that DNA methylation is depicted as residuals which are centered at 0. FBB-ADHS: German ADHD rating scale.

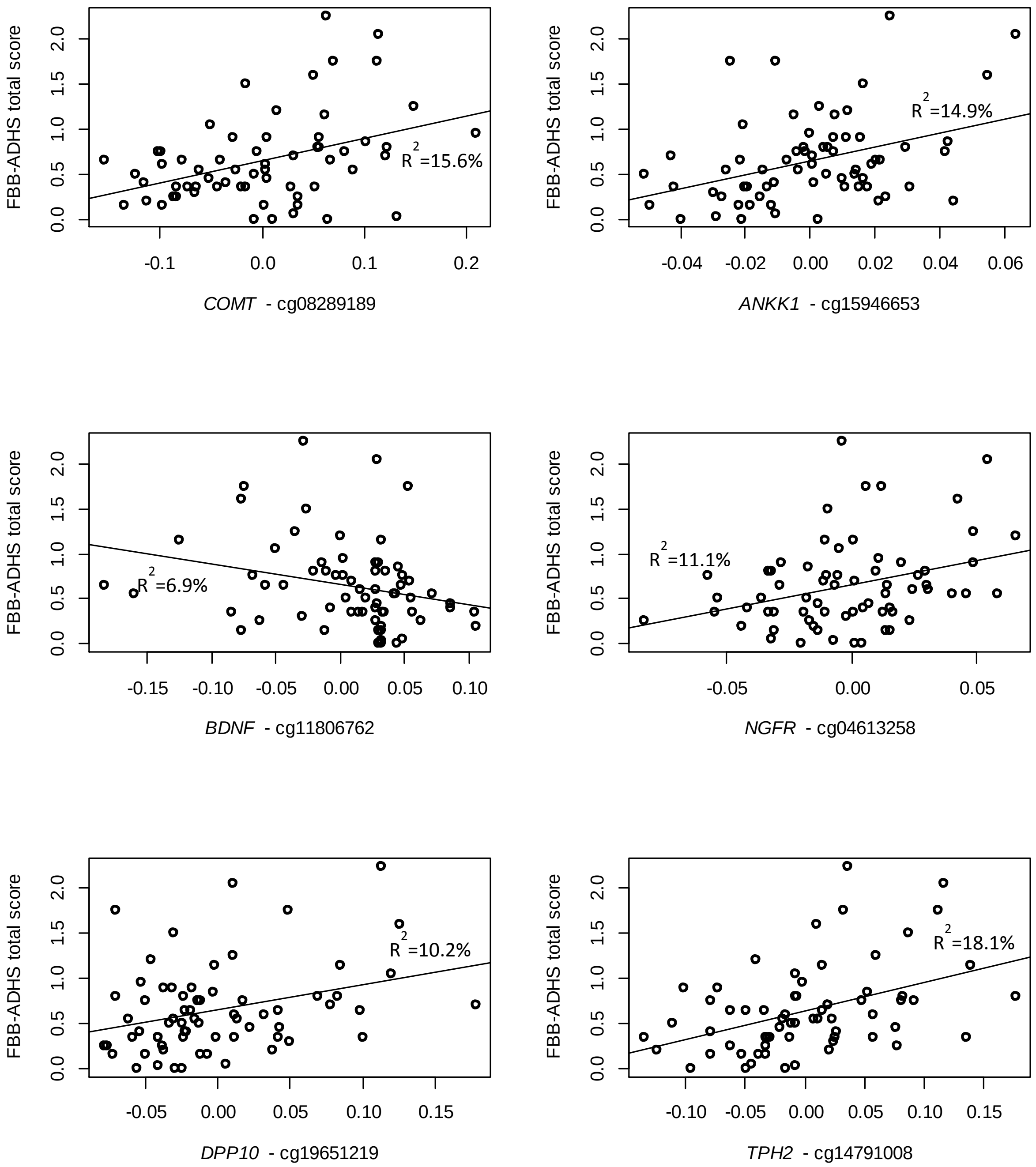

Figure S-2. Principal component analysis (PCA) before and after control probe adjustment. Technical bias (regarding plate and chip) is clearly reduced. R^2 is depicted (coded by colour and circle size). The corrplot package was used to generate the figure.

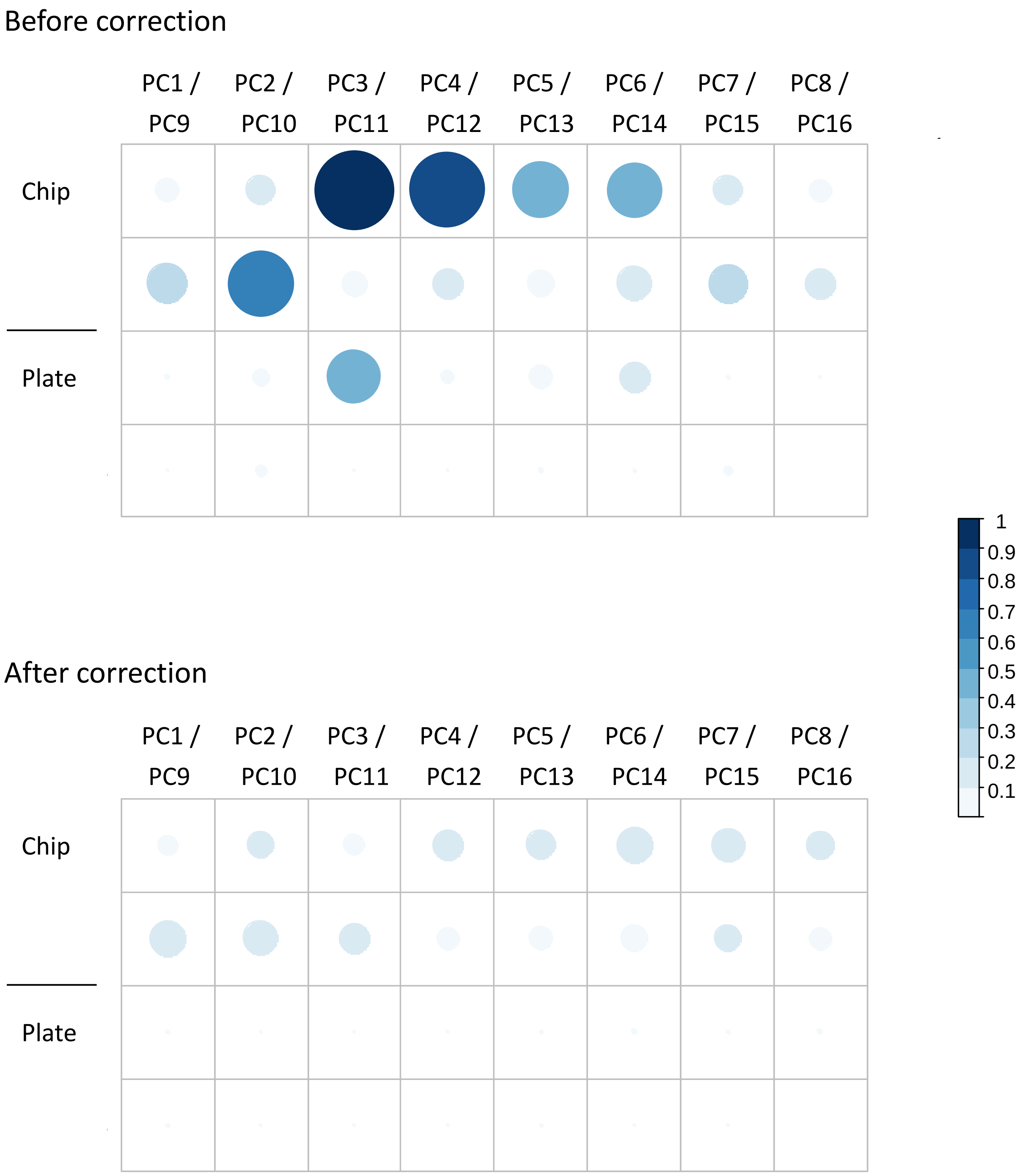

Table S-5: Candidate genes and number of CpGs

60 candidate genes (comprising 2031 CpGs) were included in the analysis. All CpGs linked to those candidate genes as defined in the HumanMethylation450K Manifest File (v.1.2) except probes containing SNPs and virtually showing no variance (i.e., mean beta values below 0.01 or above 0.99 were considered.

Genes	no. CpGs	Genes	no. CpGs
DRD1	8	*NTRK2*	14
DRD2	16	*NTRK3*	30
DRD3	9	*CDH13*	51
DRD4	17	*GFOD1*	38
DRD5	12	*MTA3*	13
DAT1/SLC6A3	45	*SPATA13*	42
COMT	29	*UNC5B*	26
ANKK1	10	*ASTN2*	24
DDC	34	*CSMD2*	68
DBH	8	*ITGAE*	32
NET1/SLC6A2	16	*ITGA11*	28
ADRA2A	11	*CDH23*	98
ADRA2C	9	*GPC6*	63
ADRA1A	17	*CTNNA2*	76
ADRA1B	17	*NAV2*	114
ADRB1	7	*KCNIP4*	45
ADRB2	8	*KCNIP1*	35
SLC6A4	15	*DPP10*	45
HTR1B	10	*FHIT*	55
HTR2A	21	*KCNC1*	26
TPH2	19	*SNAP25*	16
BDNF	50	*CHRNA4*	18
NGF	23	*SLC9A9*	20
NGFR	24	*SUCNR1*	21
NTF3	36	*NOS1*	20
NTF4	6	*GRIN1*	31
CNTF	23	*GRIN2A, GRIN2B, GRIN2C, GRIN2D*	134
CNTFR	12	*GRIN3A,GRIN3B*	25
GDNF	39	*MYT1L*	155
NTRK1	46	*VIPR2*	71

Table S-6: Sample characteristics for the three groups of boys classified along their FBB-ADHS total score: controls (≤ 0.5); ADHD-low: (> 0.5 / ≤1); ADHD-high (> 1). The groups did not differ significantly regarding age, IQ, socioeconomic status and absence or presence of prenatal maternal smoking and prenatal maternal depressive symptomatology. There was a tendency regarding prenatal alcohol exposure (higher probability in the ADHD-high group).

	Controls	ADHD-low	ADHD-high	Statistics
N	40	29	13	
Age (years)	7.58 ± 0.52	7.52 ± 0.68	7.45 ± 0.43	$F(2,79) = 0.24$, n.s.
IQ	105.4 ± 8.1	104.5 ± 11.6	101.3 ± 10.7	$F(2,79) = 0.87$, n.s.
Socioeconomic status	11.6 ± 1.8	11.5 ± 2.0	10.5 ± 2.3	$F(2,79) = 1.59$, n.s.
ADHD rating scale - total score	0.29 ± 0.14	0.72 ± 0.12	1.55 ± 0.39	$F(2,79) = 207.9$, $p<0.001$
Prenatal risk factors:				
Maternal smoking	3 (7.5%)	7 (24.1%)	2 (15.4%)	$\chi^2 = 3.73$, p = n.s.
Alcohol exposure	7 (17.5%)	5 (17.2%)	6 (46.2%)	$\chi^2 = 5.28$, $p = 0.07$
Maternal depressive symptoms	5 (12.5%)	4 (13.8%)	3 (23.1%)	$\chi^2 = 0.90$, n.s.
